# Advances in Perovskite Solar Cells

**DOI:** 10.1002/advs.201500324

**Published:** 2016-01-21

**Authors:** Chuantian Zuo, Henk J. Bolink, Hongwei Han, Jinsong Huang, David Cahen, Liming Ding

**Affiliations:** ^1^ National Center for Nanoscience and TechnologyBeijing100190P.R. China; ^2^ Department of Materials and InterfacesWeizmann Institute of ScienceRehovot76100Israel; ^3^ Instituto de Ciencia MolecularUniversidad de ValenciaValencia46022Spain; ^4^ Michael Grätzel Center for Mesoscopic Solar CellsHuazhong University of Science and TechnologyWuhan430074P.R. China; ^5^ Department of Mechanical and Materials EngineeringUniversity of Nebraska‐LincolnLincolnNE68588USA

**Keywords:** applications, device structures, perovskite solar cells

## Abstract

Organolead halide perovskite materials possess a combination of remarkable optoelectronic properties, such as steep optical absorption edge and high absorption coefficients, long charge carrier diffusion lengths and lifetimes. Taken together with the ability for low temperature preparation, also from solution, perovskite‐based devices, especially photovoltaic (PV) cells have been studied intensively, with remarkable progress in performance, over the past few years. The combination of high efficiency, low cost and additional (non‐PV) applications provides great potential for commercialization. Performance and applications of perovskite solar cells often correlate with their device structures. Many innovative device structures were developed, aiming at large‐scale fabrication, reducing fabrication cost, enhancing the power conversion efficiency and thus broadening potential future applications. This review summarizes typical structures of perovskite solar cells and comments on novel device structures. The applications of perovskite solar cells are discussed.

## 1 Introduction

Solar energy is clean and nearly inexhaustible. To harvest solar energy is a promising approach to solve the energy problem of human beings. Photovoltaic (PV) cells can be an effective way to convert solar energy directly into electricity without any moving parts. Solar cells based on crystalline silicon are by now widely commercialized, but possiblities to increase their performance are limited, because nowadays the so‐called balance of systems (BoS) cost of PV modules makes up most of the cost.^1^ As much of the BoS scales with area, performance improvement seems the only way to decrease the cost of PV power further. Therefore, new PV cells are sought either to allow for higher efficiencies than what is possible with Si PV without cost increase, or, as explained in section 3.4, to provide low‐cost added efficiency to Si PV. Emerging solar cells such as dye‐sensitized, bulk‐heterojunction and quantum‐dot solar cells can be fabricated via low‐temperature solution processing, that holds promise for low‐cost large scale application, but best power conversion efficiencies (PCE) are half of, or less than that of the best commercial Si PV cells.^2^ This is where perovskite solar cells enter, as for the first time in PV history it is possible to produce high‐efficiency cells at low monetary and energy costs, with apparent ease of fabrication from earth‐abundant, readily available raw materials. The PCE for perovskite solar cells has increased from 2.2% to 20.1% since 2006, showing an inviting vista of commercialization.^3^, ^4^

Perovskite solar cells are named after the crystal structure of the light absorbers, the structure of the mineral CaTiO_3_. Many compounds with ABX_3_ stoichiometry take this structure, where A and B are 12‐ and 8‐coordinates cations, respectively, and X is the anion.^4^ Of the many ABX_3_ only few are suitable to be efficient light absorbers for solar cells due to requirements such as appropriate bandgap for good light‐harvesting ability, energy level/band alignment with contacting materials, long charge carrier lifetime, τ, and high mobility, μ. Perovskites generally have divalent anions, and the strong electrostatic bonding mostly makes their (high) bandgaps not suitable for solar PV. Mitzi et al. initiated using perovskites containing halides, ammonium cations and Sn^2+^ in optoelectronic devices, which formed the basis for the development of perovskites for solar cells.^5^ Here we will focus on such halide perovskites, together with one divalent (Pb^2+^) and one monovalent (mostly CH_3_NH_3_^+^) cation. The most efficient halide perovskite solar absorbers consist of organic ammonium ions (CH_3_NH_3_^+^ or NH = CHNH_3_^+^), Pb^2+^ and halide ions (I^−^, Br^−^).^4^ CH_3_NH_3_PbI_3_ possesses broad and intense light absorption. It is an ambipolar semiconductor (can be n‐ or p‐type), and its charge carriers can have long diffusion lengths and lifetimes, which allow excellent PCE for solar cells made with it.^3^, ^4^, ^5^, ^6^ Another advantage of perovskite absorbers is the low‐temperature solution‐processing ability, which helps to reduce fabrication cost. While other optoelectronic devices based on lead halide perovskite materials have been made, such as lasers,^7^ photodetectors^8^ and light emitting diodes,^9^ solar cells based on these materials are the most widely studied devices.

Here, we summarize the important progress of perovskite solar cells by reviewing much of the milestone work since 2006. Key factors influencing device performance are discussed. Perovskites started out as light absorbers in “dye‐sensitized” cells, but are nowadays nearly exclusively studied as absorbers in solid state, thin film‐like PV cell structures. Numerous cell types spanning a variety of device architectures have been reported. Further innovation may help to develop high performance devices and to explore new applications. At the same time, some problems need to be tackled and solved or circumvented, including the origin of hysteresis in current–voltage curves, and concomitant issues on experimentally measured efficiencies and, more generally, electrical performances that are reported; reproducibility of sample and device preparations, properties and performance; and up‐scaling.

## 2 Common Structures for Perovskite Solar Cells

### 2.1 Liquid‐Electrolyte Dye‐Sensitized Cells

**Figure 1:**
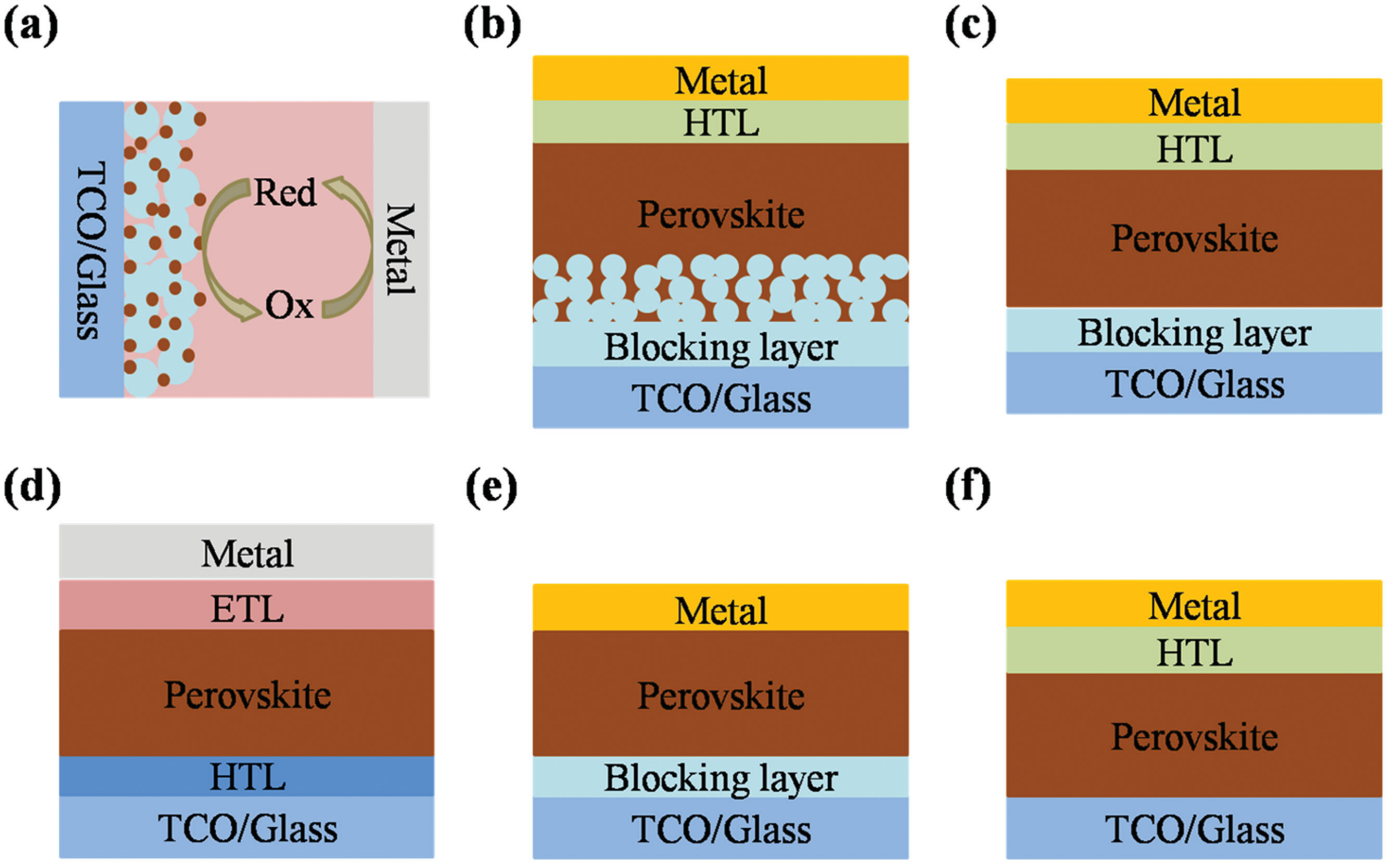
Typical structures for perovskite solar cells. TCO: transparent conducting oxide; HTL: hole transport layer; ETL: electron transport layer.

These cells consist of a transparent conducting oxide (TCO) substrate, nanoporous TiO_2_, a perovskite sensitizer, an electrolyte and a metal counter electrode (**Figure** **1**a). CH_3_NH_3_PbBr_3_ was first used as the sensitizer for TiO_2_ in dye‐sensitized solar cells, and the cells gave a PCE of 2.2%.^10^ When CH_3_NH_3_PbI_3_ was used as the sensitizer, a 3.8% PCE was achieved.^11^ The lower bandgap and wider absorption spectrum of the iodide absorber led to an enhanced short‐circuit current density (*J*_sc_). A 6.5% PCE was achieved via optimizing the preparation of CH_3_NH_3_PbI_3_ and TiO_2_ nanoparticles.^12^ Research on liquid‐electrolyte dye‐sensitized cells did not continue because these cells are highly unstable (80% decrease in PCE in 10 min) and no suitable liquid electrolyte was found in which the absorber was stable. We note that a battery effect might have been involved in these cells, which could increase output power, as a result of the free energy gain from a chemical reaction.

### 2.2 Mesoporous Structure

Kim et al. successfully fabricated first solid‐state perovskite solar cells using a solid‐state hole transport material (HTM) called 2,2′,7,7′‐tetrakis(N,N‐di‐p‐methoxyphenylamine)‐9,9′‐spirobifluorene (spiro‐OMeTAD).^13^ A 9.7% PCE was obtained by using the CH_3_NH_3_PbI_3_ absorber and a mesoporous TiO_2_ scaffold. Meanwhile, Lee et al. achieved a 10.9% PCE by using what was thought to be a mixed‐halide perovskite absorber (CH_3_NH_3_PbI_3–*x*_Cl*_x_*) (It is not clear if there is a stoichiometric amount of Cl present in these films. While throughout this report we will use the formula CH_3_NH_3_PbI_3–*x*_Cl*_x_*, likely often only traces of Cl are present, which in the literature is at times reflected by CH_3_NH_3_PbI_3_(Cl)) and Al_2_O_3_ scaffold.^14^ The results indicated that the cell need not be a sensitized one as injection of electrons into the wide bandgap Al_2_O_3_ is not possible, and also, that both electrons and holes can be transported in the perovskite. The absence of the liquid electrolyte significantly improved the stability of the devices.^13^, ^14^

The cells with mesoporous structure generally consist of a TCO (FTO or ITO), a hole blocking layer, a mesoporous TiO_2_ or Al_2_O_3_ scaffold, the perovskite absorber, a hole transport layer (HTL) and the metal electrode (Figure 1b). The morphology (surface coverage, grain size and uniformity, roughness, etc.) for the perovskite layer affects device performance significantly.[[qv: 3d]] CH_3_NH_3_PbI_3_ films with a better morphology were obtained by using a two‐step deposition method. Dipping a TiO_2_/PbI_2_ composite film into a 2‐propanol solution of CH_3_NH_3_I led to the in situ formation of CH_3_NH_3_PbI_3_. Using this approach to prepare solar cells led to a 15.0% PCE and an improved reproducibility.^15^ Jeon et al. improved the uniformity and density of the perovskite layers by solvent engineering, and achieved a certified PCE of 16.2% and improved stability for CH_3_NH_3_Pb(I_1–*x*_Br*_x_*)_3_ (*x* = 0.1–0.15) cells.^16^ Im et al. developed a two‐step spin‐coating procedure to prepare CH_3_NH_3_PbI_3_ cuboids with controlled size and achieved a PCE of 17.0%.^17^ In a further development, Jeon et al. stabilized the crystal structure of formamidinium lead iodide (FAPbI_3_), which has a lower bandgap than that of CH_3_NH_3_PbI_3_, by incorporating methylammonium lead bromide (MAPbBr_3_), achieving a certified PCE of 17.9%.^18^ Recently, they achieved a certified PCE of 20.1% from FAPbI_3_ cells prepared via an intramolecular exchange method.^19^ It should be noted that all these high efficiency values were obtained from small cells (<1 cm^2^), and that it is not always clear if hysteresis was considered when reporting the photocurrent, photovoltage, fill factor and PCE of a cell.^20^

Besides a high efficiency, the cost and stability should be of concern for the commercialization of perovskite solar cells. Various HTMs were developed to replace the expensive spiro‐OMeTAD. Now polytriarylamine (PTAA) is the most efficient organic HTM, but it needs dopants like Li‐bis(trifluoromethanesulfonyl)imide (Li‐TFSI) or 4‐*tert*‐butylpyridine (TBP).^19^ These dopants harm the device stability.^21^ Some dopant‐free organic HTMs showed good performance. Using pristine tetrathiafulvalene derivative TTF‐1 as HTM, solar cells gave a PCE of 11.0%.[[qv: 21a]] Using P3HT/graphdiyne composite as HTM, solar cells gave a PCE of 14.6%.[[qv: 21b]] Some inorganic HTMs were developed. Copper iodide (CuI) and copper thiocyanate (CuSCN) were used as HTMs to replace spiro‐OMeTAD and the cells gave PCEs of 6.0% and 12.4%, respectively.^22^

### 2.3 Planar n‐i‐p Structure

The planar heterojunction structure refers to the cell structure without mesoporous scaffold. We define a cell structure as an n‐i‐p or p‐i‐n one, based on the sequence of functional layers in the device *starting from the layer onto which light is incident*. The general structure for planar n‐i‐p perovskite solar cells is shown in Figure 1c. Compact TiO_2_ or ZnO films are often used as hole blocking layers or electron‐transport layers (ETLs). Ball et al. first reported perovskite solar cells with a planar structure of FTO/compact TiO_2_/perovskite/spiro‐OMeTAD/Au.^23^ The same group (Eperon et al.) then reported planar heterojunction perovskite solar cells with an 11.4% PCE by optimizing the processing conditions (atmosphere, annealing temperature, film thickness).^24^ Using a dual‐source vapor deposition method to prepare CH_3_NH_3_PbI_3–*x*_Cl*_x_* film led to an improved PCE of 15.4%.^25^ There is a large difference in morphology between the perovskite films prepared by dual‐source vapor deposition method and one‐step solution processing method. The film prepared by the dual‐source vapor deposition method is extremely uniform and smooth, at least over ≈0.1 cm^2^ cell area. To simplify the preparation of perovskite film while keeping high film quality, Liu and Kelly used a sequential deposition method to prepare CH_3_NH_3_PbI_3_ film. Using this method and using low‐temperature solution‐processed ZnO as ETL, a 15.7% PCE was achieved.^26^ This low‐temperature fabrication method can reduce the fabrication cost and is compatible with polymer substrates.

The performance of planar heterojunction perovskite solar cells was further improved by using new electron/hole transport materials, which can improve perovskite film quality and facilitate charge extraction. Using yttrium‐doped TiO_2_ (Y‐TiO_2_) as ETL and annealing the CH_3_NH_3_PbI_3_ films in an atmosphere with 30 ± 5% relative humidity led to reduced charge recombination and facilitated charge extraction; solar cells made via this approach achieved a PCE of 19.3%.^27^ Embedding Au nanoparticles in TiO*_x_* to form a TiO*_x_*–Au–TiO*_x_* composite layer was reported to enhance charge extraction, yielding a 16.2% PCE.^28^ Using SnO_2_ as ETL, solar cells gave a PCE of 18.1% from the forward scan and a PCE of 18.4% from the reverse scan.^29^ Dopant‐free HTMs were also developed for planar heterojunction perovskite solar cells. A conjugated small molecule DOR3T‐TBDT was used as dopant‐free HTM and the solar cell gave a PCE of 14.9%.^30^ Developing novel electron/hole transport materials for perovskite solar cells may help to reduce fabrication cost and improve device stability for future commercialization.

### 2.4 Planar p‐i‐n Structure

The difference between the p‐i‐n structure and the n‐i‐p structure is the relative location of charge transport layers (Figure 1d). For the p‐i‐n structure, the HTL is on top of the transparent conducting substrate. An often‐used combination of hole and electron transporting layers in the p‐i‐n structure is poly(3,4‐ethylenedioxythiophene):polystyrene sulfonate (PEDOT:PSS) as HTL and a fullerene derivative, e.g., [6,6]‐phenyl‐C_61_‐butyric acid methyl ester (PC_61_BM) or [6,6]‐phenyl‐C_71_‐butyric acid methyl ester (PC_71_BM) as ETL. Solar cells with the p‐i‐n structure have advantages over n‐i‐p ones because of the possibility of low‐temperature preparation, of foregoing the need of dopants in the HTL and compatibility with organic electronics manufacturing processes.

The first p‐i‐n perovskite solar cell reported by Guo et al. gave a PCE of 3.9%.^31^ The cells were made by thermally depositing C_60_, bathocuproine (BCP) and Al sequentially onto ITO/PEDOT:PSS/CH_3_NH_3_PbI_3_ substrate. Lam et al. developed solution‐processed perovskite solar cells with a structure of ITO/PEDOT:PSS/CH_3_NH_3_PbI_3_/PC_61_BM/Al and obtained a 5.2% PCE by using one‐step deposition method and a 7.4% PCE by using a sequential deposition method.^32^ The PCE was improved to 12% when using CH_3_NH_3_PbI_3_ prepared by co‐evaporation of CH_3_NH_3_I and PbI_2_.^33^ Docampo et al. reported solution‐processed CH_3_NH_3_PbI_3–*x*_Cl*_x_*‐based cells with a 9.8% PCE.^34^ Then You et al. improved the PCE of the cells made by this method to 11.5% by optimizing the device preparation (device structure, thermal annealing, etc.).^35^ Wang et al. applied two fullerene layers for electron transporting to passivate the charge traps at grain boundaries, yielding an efficiency of 12.8%. The fill factor (FF) of these devices exceeded 80% for the first time in perovskite solar cells. It was also found for the first time that an optimized MAI:PbI_2_ ratio of precursor was not 1 when using the one‐step spin‐coating method, as a MAI‐rich precursor gave better morphology and PCE.^36^

The challenge in fabricating planar p‐i‐n cells on a flat TCO electrode is to obtain a smooth, pinhole‐free perovskite film to avoid leakage current by the one‐step spin‐coating method. The cells made from a PbCl_2_/CH_3_NH_3_I solution (molar ratio 1:3) showed better performance than the cells made from a PbI_2_/CH_3_NH_3_I solution (molar ratio 1:1) since the latter perovskite films showed a rough surface and a low crystallinity, also after annealing at ≈100 ºC, when no scaffold was used.^35^, ^36^, ^37^ Indeed, it is likely that one of the big advantages of the presence of a mesoporous scaffold is that it facilitates conformal, continuous coverage of the absorber that fills its pores.[[qv: 3d]] Adding CH_3_NH_3_Cl or NH_4_Cl to the PbI_2_/CH_3_NH_3_I solution improved the film morphology and increased the crystallinity, leading to a better device performance.^37^ When using the NH_4_Cl additive, good morphology and crystallinity were obtained, and the PCE increased from <0.1% to 9.9% and the FF exceeded 80%.[[qv: 37a]] These results indicate that the morphology and crystallinity of the perovskite layer are crucial to the device performance. Huang et al. developed a two‐step interdiffusion method, which is similar to the two‐step method first introduced by Burschka et al., while it was modified to involve a thermal annealing process, combined with orthogonal solvents for the two‐step spin‐coating process. It produced continuous, pinhole‐free ≈0.1 cm^2^ perovskite films on flat surface without TiO_2_, resulting in a best efficiency of 15.4% (no photocurrent hysteresis) with PEDOT:PSS as HTL, with 90% of the devices giving efficiency above 14.5%.^38^

The grain size affects PCE significantly in some devices, and it has been argued that it determines charge recombination at grain boundaries. We note, though, that Edri et al. and others showed <30 mV potential discontinuities between grains, i.e., less than or comparable to the thermal energy at the cells' operating temperatures.^39^ Xiao et al. used a solvent‐annealing method to grow perovskite films with large grain size, getting a PCE of 15.6%.^40^ Nie et al. prepared pinhole‐free perovskite films with mm‐scale crystals by using a hot casting technique, and the “millimeter” single crystals helped to boost the PCE to 17.7%.^41^ Adding hydriodic acid into CH_3_NH_3_PbI_3_ precursor solution led to dense and pinhole‐free perovskite films and an 18.1% PCE.^42^ Using a non‐wetting PTAA HTL, Huang et al. grew very large grains with aspect ratios of ≈2.3–7.9, enhancing the stabilized PCE to 18.3%.[[qv: 43a]] By incorporating Cl in the precursor, an abnormal grain‐growth behavior was observed in a multi‐cycle solution coating process, and large grains were found in perovskite films. An 18.9% PCE was achieved, which is the highest PCE at the time of writing for p‐i‐n perovskite solar cells.[[qv: 43b]]

New interlayers were developed to improve electron transport between ETL and metal electrode.^44^ A 14.1% PCE was obtained by using a 0.5 nm LiF interlayer, much higher than the PCE of the device without LiF (11.5%). A 10 × 10 cm^2^ module with an 8.7% PCE was made by using a LiF interlayer.[[qv: 44a]] Inserting an interlayer of an amino‐functionalized polymer, PN4N, between PC_61_BM and Al increased the PCE from 12.4% to 15.0%.[[qv: 44b]] Poly[(9,9‐bis(3′‐(N,N‐dimethylamino)propyl)‐2,7‐fluorene)‐alt‐2,7‐(9,9‐dioctyl fluorene)] (PFN) also facilitated electron extraction from PCBM to Al, giving a 17.1% PCE.[[qv: 44c]] Several other groups have made significant efforts in large area cells and modules, but, as most of these research is commercial, only little is published.^45^

Some inorganic HTMs were developed to improve the device stability. A 7.8% PCE was achieved by using NiO*_x_* as HTM.[[qv: 46a]] Doping NiO*_x_* with Cu improved the conductivity of NiO*_x_*, leading to a 15.4% PCE and good stability.[[qv: 46b]] Using NiO deposited by a pulsed laser deposition (PLD) method yielded a PCE of 17.3%.[[qv: 46c]] PCEs of 12.2% and 13.4% were achieved by using CuO and Cu_2_O as HTMs, respectively.[[qv: 47a]] Electrodeposited CuSCN was used as the HTM for p‐i‐n perovskite solar cells and a 16.6% PCE was obtained.[[qv: 47b]]

### 2.5 HTL‐Free Cells

HTL‐free perovskite solar cells were made by directly depositing Au onto the perovskite layer without using a HTL (Figure 1e). While unlikely to be of practical interest because of the slow chemical reaction between Au and iodide, such cells are of great interest to understand work mechanism of perovskite solar cells. Here the perovskite acts as an absorber and as hole conductor, forming a heterojunction with ETL like TiO_2_. The built‐in field drives the separation of charge carriers. The first HTL‐free perovskite solar cells gave a PCE of 5.5%.^48^ Shi et al. got a 10.5% PCE by using a two‐step deposition method and argued that their TiO_2_/CH_3_NH_3_PbI_3_/Au cell is a typical heterojunction solar cell.^49^ When they inserted an ultrathin Al_2_O_3_ film between CH_3_NH_3_PbI_3_ and Au to block electrons, they achieved an 11.1% PCE.^50^ Two HTL‐free cells with structures of ITO/CH_3_NH_3_PbI_3_/PC_61_BM/Bis‐C_60_/Ag^51^ and ITO/CH_3_NH_3_PbI_3_/C_60_/BCP/Ag^52^ (BCP = 2,9‐dimethyl‐4,7‐diphenyl‐1,10‐phenanthroline) were reported, giving PCEs of 11.0% and 16.0%, respectively. Bis‐C_60_ and BCP were used to block holes and facilitate electron transport.^36^, ^51^

### 2.6 ETL‐Free Cells

TiO_2_ and other n‐type semiconductors as ETL were thought necessary to make perovskite solar cells. However, high PCEs can also be achieved without using such ETL (Figure 1f). Liu et al. deposited CH_3_NH_3_PbI_3_ directly onto ITO by using a sequential deposition method and achieved a 13.5% PCE.^53^ Ke et al. reported ETL‐free cells with a 14.1% PCE by directly forming CH_3_NH_3_PbI_3–*x*_Cl*_x_* film on FTO.^54^ They suggested that the key for obtaining efficient ETL‐free cells is to prepare uniform perovskite films with good crystallinity, avoiding shunting paths between HTL and FTO. Some “ETL‐free” cells exhibited very low stabilized power output even though decent PCEs were obtained from *J–V* measurements.^55^ Therefore, the working mechanism for these cells needs further investigation. We note that ITO and FTO behave as ETL, thus the term “ETL‐free” should be taken with a grain of salt.

### 2.7 Further Investigations

The structure diversity for perovskite solar cells correlates with the outstanding optoelectronic properties of perovskite materials. The exciton binding energy for CH_3_NH_3_PbI_3_ was estimated to be ≈2–50 meV, and, de facto, at room temperature under solar illumination, the thermal energy suffices for the excitons to dissociate into free charge carriers.^56^ Electron/hole diffusion lengths were found to be >1 μm in CH_3_NH_3_PbI_3–*x*_Cl*_x_* films.^6^, ^57^ >175 μm diffusion lengths in single crystals of CH_3_NH_3_PbI_3_ may result from intrinsic character of the material (i.e., the distinction in diffusion length between minority and majority carriers is blurred in such systems).^58^ Hole mobilities of 164 ± 25 cm^2^ V^−1^ s^−1^ and electron mobilities of 24.8 ± 4.1 cm^2^ V^−1^ s^−1^ were determined for CH_3_NH_3_PbI_3_ single crystals using the space charge limited current (SCLC) method, in agreement with results from Hall effect and time‐of‐flight (ToF) measurements.^58^ The long electron/hole diffusion lengths and good mobilities allow efficient collection of the free charge carriers by the electrodes in the thin planar‐structure cells. With further, more systematic investigations, perovskite solar cells, and, especially modules, should be able to evolve further. Some research areas are:

- device components (which is likely to yield improved understanding of mechanisms), materials stability, and device performance reproducibility;
- additional areas ripe for further study are charge transport, interface engineering, to achieve control over wetting (important to make reproducibly large area devices), and energy level/band alignment;
- novel materials development;
- advanced fabrication technologies;^59^
- for the commercial development of solar cells, it will be great if a clear winner in terms of device structure emerges soon, so as to make cell development more CdTe‐ and less CIGS‐like.

## 3 Advanced Structures for Perovskite Solar Cells

### 3.1 Flexible Cells

**Figure 2:**
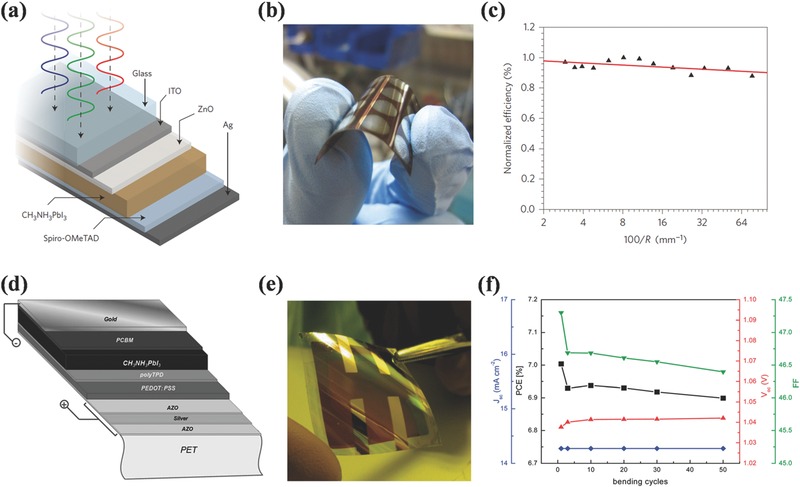
a) Structure for an ITO/ZnO/CH_3_NH_3_PbI_3_/spiro‐OMeTAD/Ag cell on glass substrate; b) perovskite solar cells on PET substrate; c) normalized PCEs for the bent cells as a function of the radius (*R*) of bending; d) structure for a flexible perovskite solar cell; e) flexible solar cells; f) PCE, FF, *J*_sc_ and *V*_oc_ change with the bending cycles. a–c) Reproduced with permission.^26^ Copyright 2013, Macmillan Publishers Ltd. d–f) Reproduced with permission.[[qv: 33b]] Copyright 2014, Royal Society of Chemistry.

Perovskite solar cells have been made on flexible substrates to get flexible cells.^34^ The widely used flexible substrate is poly(ethylene terephthalate) (PET). High‐temperature processing should be avoided due to the low application temperature of PET. Liu et al. reported perovskite solar cells with a 15.7% PCE by using low‐temperature processed ZnO nanoparticles as ETL (**Figure** **2**a). They further made flexible cells by replacing glass substrate with PET substrate (Figure 2b). The resulting flexible cells gave a PCE of 10.2% which decreased by <15% when bending the cell (around a cylinder with radius as small as 16 mm) until the ITO/PET substrate could not recover (Figure 2c).^26^ Recently, an amorphous TiO*_x_* layer deposited by atomic layer deposition was used in flexible perovskite solar cells and a 12.2% PCE was obtained.^60^

Another type of flexible perovskite solar cells consist of PEDOT:PSS as HTL and PCBM as ETL. PEDOT:PSS is more suitable for flexible cells due to its low‐temperature preparation and good flexibility. Roldán‐Carmona et al. reported flexible perovskite solar cells based on sublimated CH_3_NH_3_PbI_3_ (Figure 2d).[[qv: 33b]] Poly[N,N′‐bis(4‐butylphenyl)‐N,N′‐bis(phenyl)benzidine] (PolyTPD) was spin‐coated onto PEDOT:PSS to better block electrons. A 7% PCE was obtained and no significant performance deterioration was observed after the cells had been bent 50 times in a row (Figure 2f). Docampo et al. and You et al. reported flexible cells based on solution‐processed CH_3_NH_3_PbI_3–*x*_Cl*_x_* with PCEs of 6.4% and 9.2%, respectively.^34^, ^35^ Poorkazem et al. carried out fatigue resistance measurements on flexible perovskite solar cells based on CH_3_NH_3_PbI_3_.^61^ They found that the drop in device performance was caused by the cracks generated in indium oxide‐based transparent electrode after bending, not caused by the perovskite layer. The flexibility for CH_3_NH_3_PbI_3_ films is good enough for roll‐to‐roll fabrication. Recently, ultrathin (3 μm), ultra‐lightweight, and highly flexible perovskite solar cells with a stabilized efficiency of 12% were made and used to power aviation models.^62^

**Figure 3:**
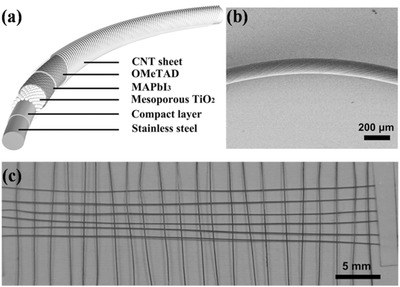
a) Structure for the fiber‐like perovskite solar cell; b) scanning electron microscope (SEM) image for a bent fiber‐like perovskite solar cell; c) a textile made with fiber‐like solar cells. Reproduced with permission.^63^

Qiu et al. fabricated fiber‐like perovskite solar cells by replacing the planar flexible substrate with a stainless steel fiber electrode and using carbon nanotube (CNT) sheets as the other electrode (**Figure** **3**a,b).^63^ The cells gave a PCE of 3.3% and could be woven to be perovskite solar cell textiles (Figure 3c). This design expands the application for perovskite solar cells. The flexibility of fiber‐like cells can be enhanced by replacing the steel fiber with a CNT fiber. The CNT fiber‐based cells worked for more than 96 h in air, giving a 3.03% PCE.^64^

### 3.2 Cells with a Carbon Electrode

**Figure 4:**
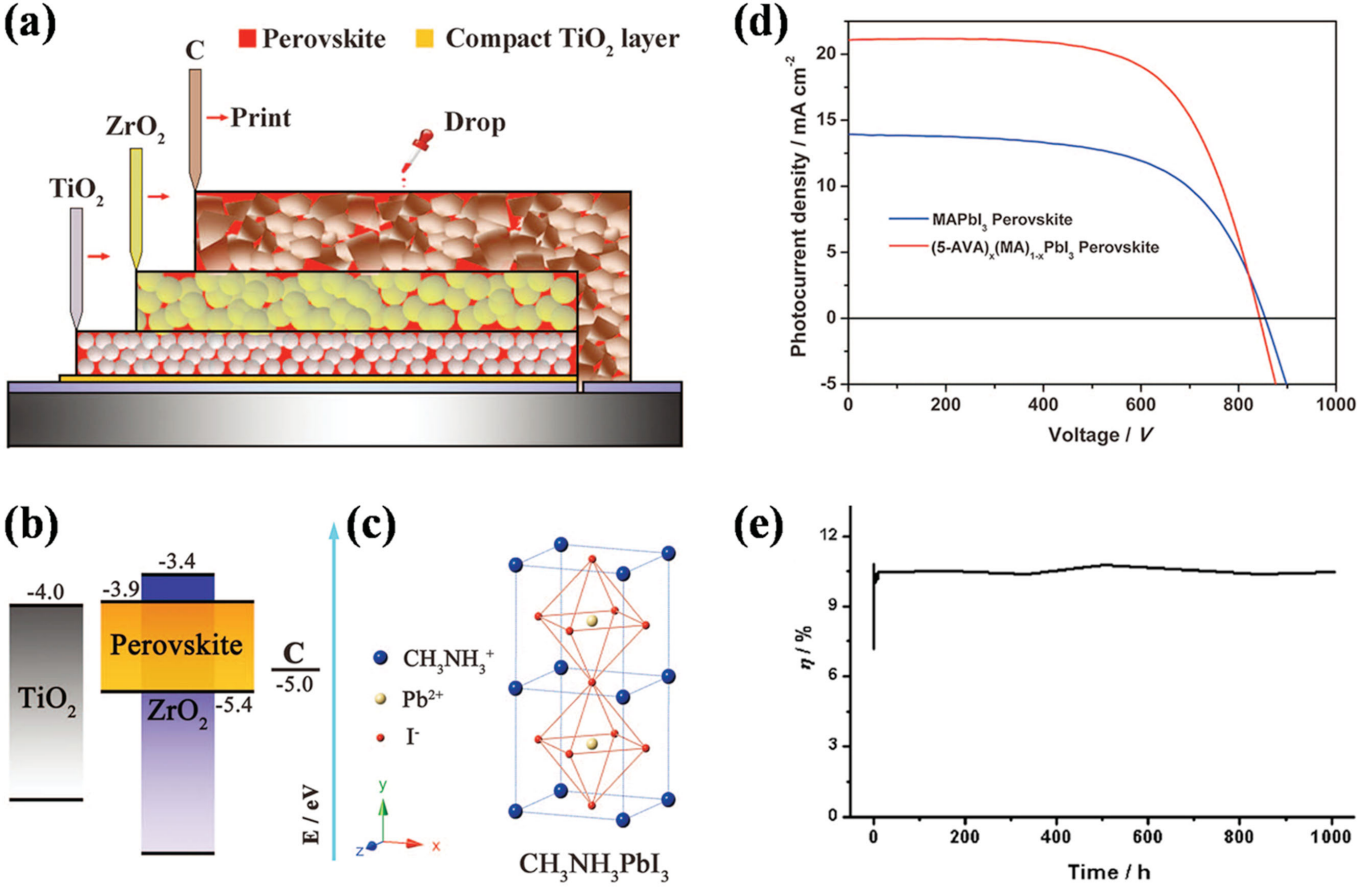
a) Fully printable mesoscopic perovskite solar cells; b) suggested energy band diagram for the device; c) crystal structure of CH_3_NH_3_PbI_3_; d) *J–V* curves for printable solar cells; e) stability test for a (5‐AVA)*_x_*(MA)_1–*x*_PbI_3_ solar cell. Reproduced with permission.^65^ Copyright 2014, American Association for the Advancement of Science.

Mei et al. developed a printing technique to fabricate mesoscopic perovskite solar cells.^65^ Nanoporous TiO_2_, ZrO_2_, and carbon black/graphite electrode were printed sequentially on TiO_2_ coated FTO glass. Then the perovskite precursor solution was dropped onto the carbon electrode, and it infiltrated through carbon electrode to reach ZrO_2_ and TiO_2_ (**Figure** **4**a). The device fabrication was finished after a thermal treatment. ZrO_2_ prevented contact between TiO_2_ and carbon electrode. The cells based on MAPbI_3_ gave a PCE of 7.2%. Introducing 5‐ammoniumvaleric acid (5‐AVA) iodide into MAPbI_3_, a mixed‐cation perovskite (5‐AVA)*_x_*(MA)_1–*x*_PbI_3_ was formed, a PCE of 11.6% and a certified PCE of 12.8% were obtained (Figure 4d). The –COOH groups of 5‐AVA anchor on the surface of mesoporous TiO_2_ and ZrO_2_ films, whereas the NH_3_^+^ groups act as nucleation sites, improving the charge transfer between TiO_2_ and perovskite. After working under 1 sun in air for more than 1000 h, the cells still gave stable PCE (Figure 4e). Introducing a self‐assembled silane monolayer between TiO_2_ and CH_3_NH_3_PbI_3_ led to a better interface and improved device performance.^66^ Effects of TiO_2_ nanoparticle size and carbon electrode composition on the device performance were systematically studied.^67^ This printing technique has potential for mass production of perovskite solar cells.

**Figure 5:**
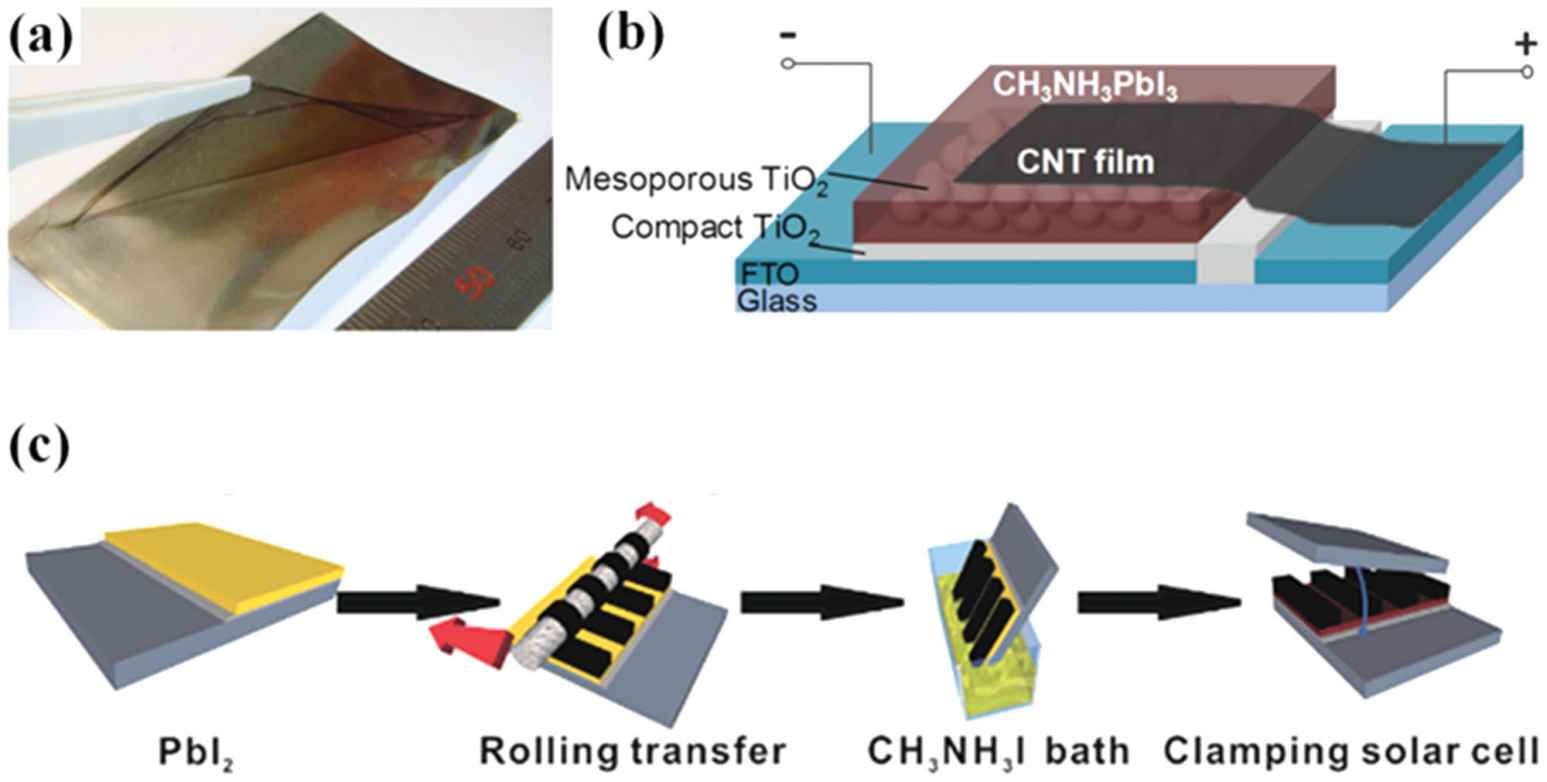
a) CNT film; b) CH_3_NH_3_PbI_3_ solar cell with CNT electrode; c) fabrication of the clamping solar cell. a,b) Reproduced with permission.^68^ Copyright 2014, American Chemical Society. c) Reproduced with permission.^69^ Copyright 2014, Royal Society of Chemistry.

Carbon electrodes can work in perovskite solar cells without HTL, and the device fabrication doesn't need vacuum evaporation, thus reducing fabrication cost. Carbon nanotubes (CNTs) have been used as electrodes in different solar cells. Li et al. used CNT electrode to make perovskite solar cells (**Figure** **5**b).^68^ The as‐prepared CNT film (Figure 5a) can be peeled off from the substrate and transferred onto the perovskite film. Toluene was dropped onto CNT film to improve the contact between CNTs and perovskite. The device fabrication was complete after toluene vaporization. Dropping spiro‐OMeTAD solution in chlorobenzene onto the CNT electrode increased PCE from 6.87% to 9.90%. Wei et al. developed a novel “clamping solar cell” using candle soot as the electrode.^69^ The cells made by directly clamping a perovskite photoanode to candle soot on FTO substrate gave a 2.60% PCE. The PCE increased to 5.44% by annealing the candle soot. Depositing a PbI_2_ layer onto TiO_2_ coated FTO glass, then transferring candle soot onto PbI_2_, and treating the substrate with CH_3_NH_3_I solution, the resulted cells gave a 11.02% PCE (Figure 5c). The PCE enhancement was attributed to the formation of an interpenetrating interface between perovskite and candle soot during in situ conversion of PbI_2_ to CH_3_NH_3_PbI_3._ Low‐temperature (100 °C) processed carbon paste was also used as electrode for perovskite solar cells, which gave an 8.3% PCE.^70^

### 3.3 Semitransparent Cells

**Figure 6:**
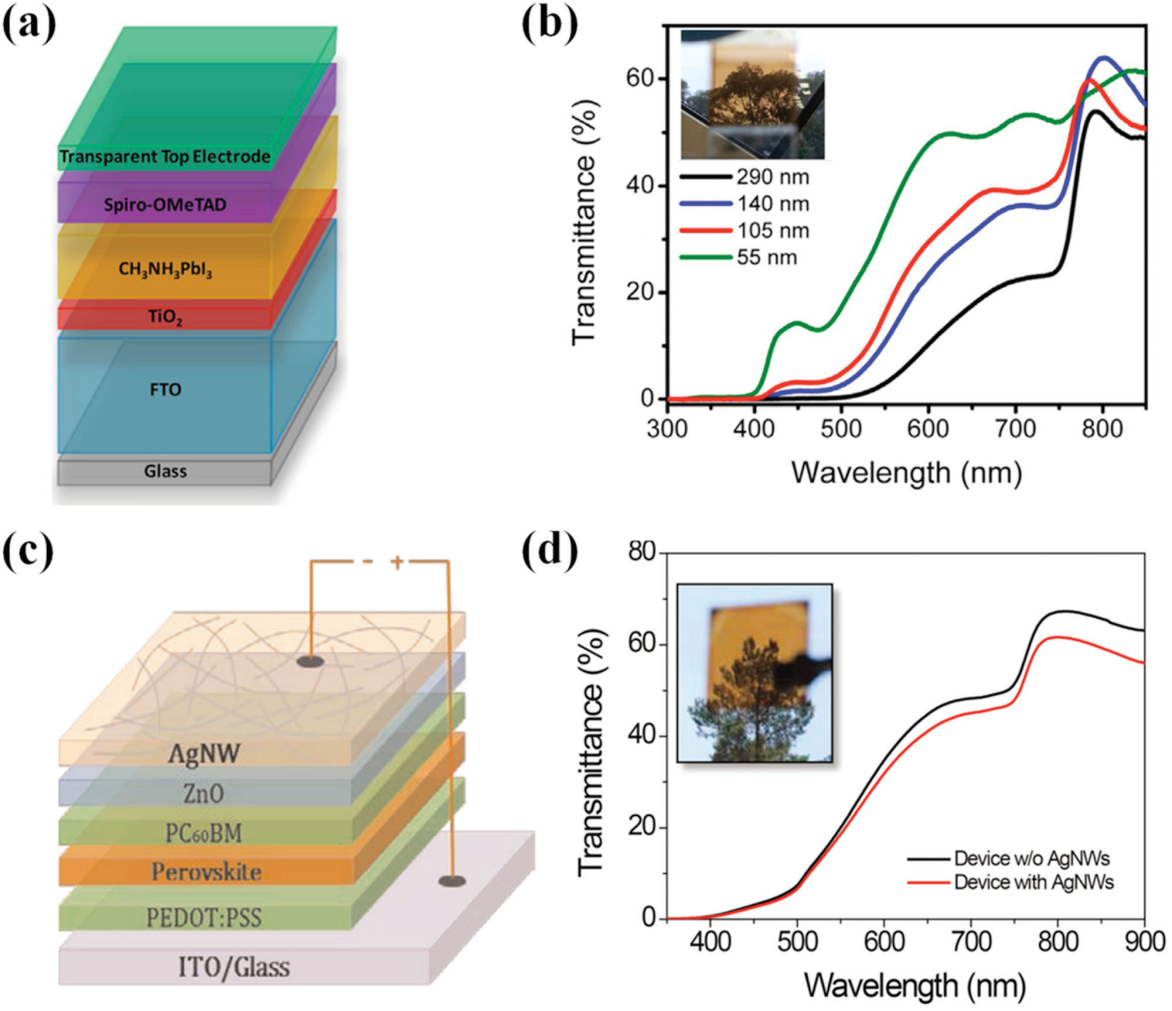
a) Structure of a semitransparent perovskite solar cell; b) transmittance spectra for semitransparent perovskite solar cells with different CH_3_NH_3_PbI_3_ layer thickness; c) structure of semitransparent perovskite solar cells with solution‐processed AgNWs electrode; d) transmittance spectra for the device before and after AgNWs deposition. a,b) Reproduced with permission.^73^ Copyright 2015, Elsevier. c,d) Reproduced with permission.^74^ Copyright 2015, Royal Society of Chemistry.

Semitransparent solar cells can find applications in windows, cladding of buildings and vehicles. The transparency of perovskite solar cells depends on the thickness of the perovskite layer and the transmittance of the electrode. A thin metal layer was often used as transparent electrode in semitransparent solar cells. Eperon et al. reported semitransparent cells with a 10 nm thick gold electrode, having an average visible transmittance (AVT) of ≈30% and a 3.5% PCE.^71^ Roldán‐Carmona et al. used a 6 nm thick gold electrode and a LiF capping layer to protect the gold layer and reduce reflection, obtaining a 6.4% PCE and a 29% AVT.^72^ Cheng et al. improved the transparency and conductivity of the transparent electrode by using a MoO_3_–Au–MoO_3_ configuration (**Figure** **6**a).^73^ MoO_3_ helped to form a thin and uniform Au layer, leading to enhanced conductivity. The thickness of CH_3_NH_3_PbI_3_ layer was adjusted to obtain different transparency (Figure 6b). PCEs of 5.3% and 13.6% were achieved for cells with AVT values of 31% and 7%, respectively.

Another type of transparent electrodes uses silver nanowires (AgNWs). Guo et al. reported semitransparent perovskite solar cells with an 8.5% PCE and a 28.4% AVT by using solution‐processed AgNWs electrode (Figure 6c).^74^ The deposition of AgNWs electrode did not affect the device transmittance much (Figure 6d). Bailie et al. reported semitransparent perovskite solar cells with AgNWs electrode giving a 12.7% PCE.^75^ The cells have a peak transmittance of 77% at 800 nm. The transmitted light was utilized in a tandem structure employing a crystalline silicon (Si) or copper indium gallium diselenide (CIGS) cell as the bottom cell, leading to an increased PCE. Recently a precious metal free transparent electrode was developed, consisting of PET, Ni mesh and conducting adhesive. Semitransparent perovskite solar cells with a 13.3% PCE were made by laminating the electrode onto CH_3_NH_3_PbI_3–*x*_Cl*_x_* coated substrate.^76^

### 3.4 Tandem Cells

The open‐circuit voltage (*V*_oc_) of a single junction solar cell is limited by *E*_g_/*q* (*E*_g_ is the bandgap of the absorber and *q* is elementary charge).^77^ For a single junction cell, the absorber with narrower bandgap cannot produce high *V*_oc_, being limited by the bandgap. The absorber with wider bandgap is eligible for producing higher *V*_oc_, but the short‐circuit current (*J*_sc_) is limited because the photons with energy lower than the absorber's bandgap do not contribute to the photocurrent.^3^, ^77^ Tandem structures can yield a solution to this problem by connecting a wide bandgap solar cell with a narrow bandgap solar cell in series. The tandem cell can absorb a broad solar spectrum and provide a high *V*_oc_, which is the sum of the *V*_oc_s of the sub‐cells. The photocurrent generated by the two sub‐cells should be balanced because the photocurrent exported from the tandem cell is limited by the sub‐cell with the smaller photocurrent.^78^

#### 3.4.1 4‐Terminal Tandem Cells

**Figure 7:**
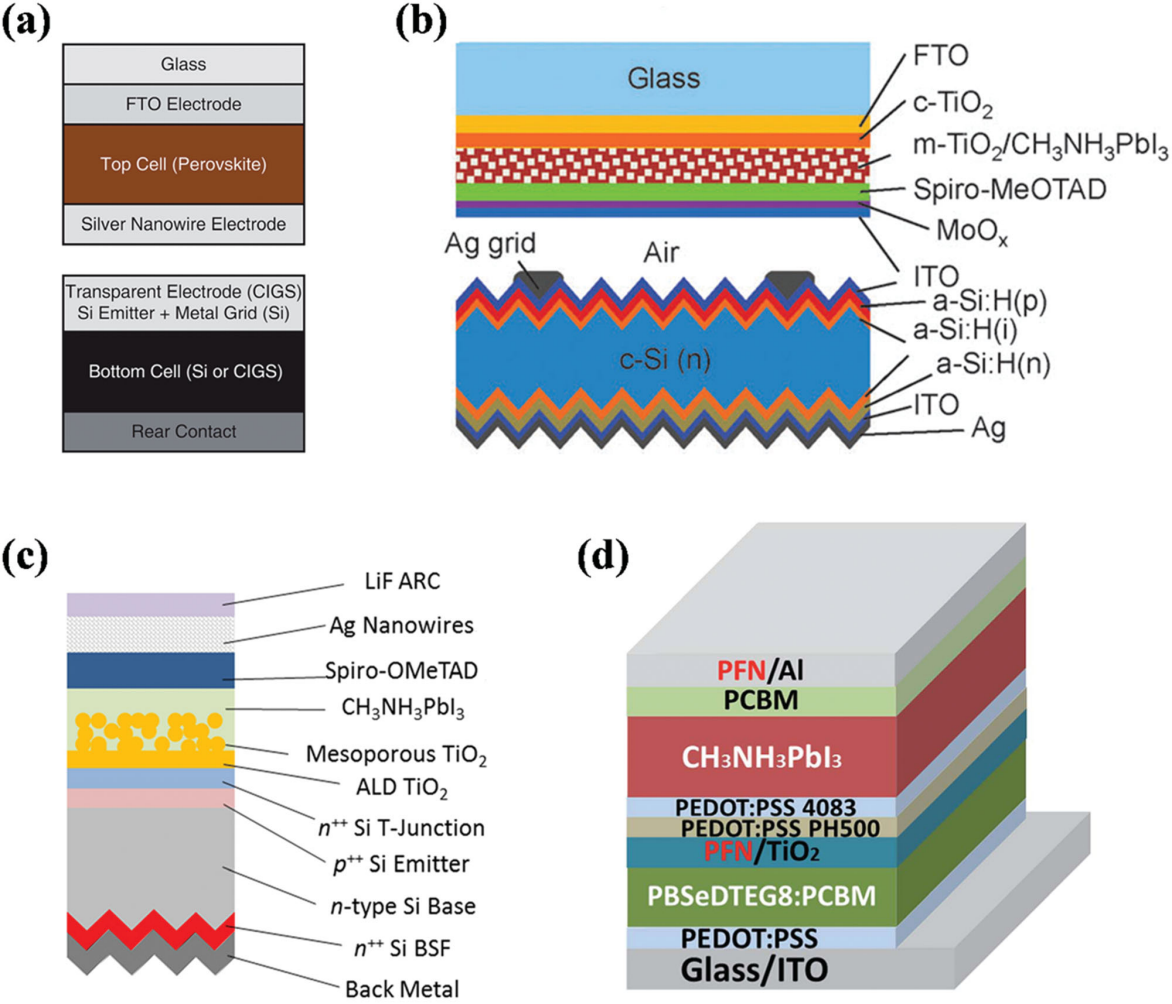
a) Structure of a mechanically stacked tandem cell with a perovskite solar cell as the top cell and a Si or CIGS cell as the bottom cell. Reproduced with permission.^75^ Copyright 2015, Royal Society of Chemistry. b) Scheme for a mechanically stacked 4‐terminal tandem cell. Reproduced with permission.^79^ Copyright 2015, Royal Society of Chemistry. c) Structure of a 2‐terminal monolithically grown perovskite/Si multi‐junction solar cell. Reproduced with permission.^80^ Copyright 2015, AIP Publishing LLC. d) Scheme of hybrid tandem solar cell, containing perovskite and polymer absorbers. Reproduced with permission.^81^ Copyright 2015, Royal Society of Chemistry.

Solar cells based on CH_3_NH_3_PbI_3_ can get much higher *V*_oc_ than Si and CIGS cells, making them suitable to be the top cells in a tandem structure.^75^ One method to make a tandem cell is to stack two sub‐cells mechanically (**Figure** **7**a,b).^75^, ^79^ Bailie et al. made a mechanically stacked tandem cell by using a semitransparent perovskite cell as the top cell and Si or CIGS cell as the bottom cell (Figure 7a).^75^ The advantage for this tandem cell is that the current matching between the top and bottom cells can be realized at the module level by adjusting the size of the sub‐cells. PCEs of 17.0% and 18.6% were obtained in CH_3_NH_3_PbI_3_/Si and CH_3_NH_3_PbI_3_/CIGS tandem cells, respectively. These PCEs were higher than those obtained from the perovskite (12.7%), Si (11.4%) and CIGS (17.0%) single cells. A maximum PCE of 30.4% can be predicted when using CH_3_NH_3_PbBrI_2_ cells in series with Si or CIGS cells.^75^ 4‐Terminal mechanically stacked tandem cells can get high PCE if the top cell can transmit the light with energy smaller than the absorber's bandgap. Naturally, and as is well known, improving the transmittance of the transparent electrode and charge transport layers can enhance the PCE of tandem cells.

#### 3.4.2 2‐Terminal Tandem Cells

Another type of tandem cells is 2‐terminal, monolithic tandem cell with an interconnection layer (Figure 7c,d).^80^, ^81^ Mailoa et al. made a monolithic tandem cell (Figure 7c) by introducing a silicon tunnel junction between the perovskite and silicon sub‐cells.^80^ The tandem cell gave a PCE of 13.7%, which is still lower than the record efficiency for perovskite or Si single junction cells. It is hard to make high efficiency sub‐cells in a tandem structure, which can work as good as the single junction cells. The tunnel junction and the current matching affect tandem cell performance significantly. Optimizing the fabrication of sub‐cells and developing better tunnel junction can improve PCE. Chen et al. fabricated a perovskite/polymer monolithic tandem cell (Figure 7d) via a low‐temperature solution processing.^81^ The relatively low PCE (10.23%) for the tandem cell resulted from the low PCE of the polymer sub‐cell.

#### 3.4.3 “All Perovskite” Tandem Cells

The previous studies focused on tandem cells consisting of high bandgap perovskite cell and another low bandgap non‐perovskite cell. Alternatively, an “all perovskite” tandem cell is possible due to the widely tunable bandgap of the Pb halide perovskite materials. The bandgap of CH_3_NH_3_Pb(I_1−_ *_x_*Br*_x_*)_3_ was reported to be tunable from 1.58 eV to 2.28 eV when *x* changes from 0 to 1,^82^ although this was later disputed and spontaneous phase separation at 0.2 < *x* < 0.8 was observed.^83^ The bandgap for HC(NH_2_)_2_Pb(I_1−_ *_x_*Br*_x_*)_3_ is in the range of ≈1.47–2.23 eV.[[qv: 82b]] A *V*_oc_ of ≈1.51 V has been obtained for a single junction cell using a CH_3_NH_3_PbBr_3–*x*_Cl*_x_* absorber.[[qv: 83c]] A *V*_oc_ of 1.06 V, a *J*_sc_ of 24.7 mA cm^−2^, a FF of 77.5% and a PCE of 20.2% were obtained by using HC(NH_2_)_2_PbI_3_ absorber.^19^ Theoretically, using HC(NH_2_)_2_PbI_3_ as the absorber for the low bandgap sub‐cell and the absorption‐complementary CH_3_NH_3_Pb(I_1−_ *_x_*Br*_x_*)_3_ as the absorber for the high bandgap sub‐cell, it is possible to get a *V*_oc_ of 2.5 V, a *J*_sc_ of 12 mA cm^−2^, a FF of 77%, leading to a PCE of 23.1% for the tandem cell. Therefore, “all perovskite” tandem solar cells might be an interesting approach to enhance the PCE of perovskite solar cells. However, there remains the challenge to prepare cells with perovskites with bandgaps of ≈1.8–2.0 eV that will not phase‐separate in operando.

### 3.5 Integrated Cells

Internal quantum efficiency (IQE) for CH_3_NH_3_PbI_3_ solar cells can reach nearly 100%,^56^, ^84^ suggesting that photocurrent loss caused by perovskite absorber itself is very small. The effective approaches to increase photocurrent are to reduce reflection, to reduce transport‐layer absorption, and to expand the photoresponse of perovskite solar cells. Some efforts have been made to reduce the bandgap of perovskite absorbers. Replacing CH_3_NH_3_^+^ in CH_3_NH_3_PbI_3_ with NH = CHNH_3_^+^ leads to a red‐shifted absorption edge (840 nm).^19^ Replacing Pb^2+^ with Sn^2+^ also leads to a narrower bandgap, but Sn^2+^ suffers from ease of being oxidized to Sn^4+^.^4^ New approaches to expand the photoresponse of perovskite solar cells were reported, i.e., integrating the cells with a bulk heterojunction layer or quantum dot layer with complementary absorption.

#### 3.5.1 Perovskite/Bulk Heterojunction Integrated Cells

**Figure 8:**
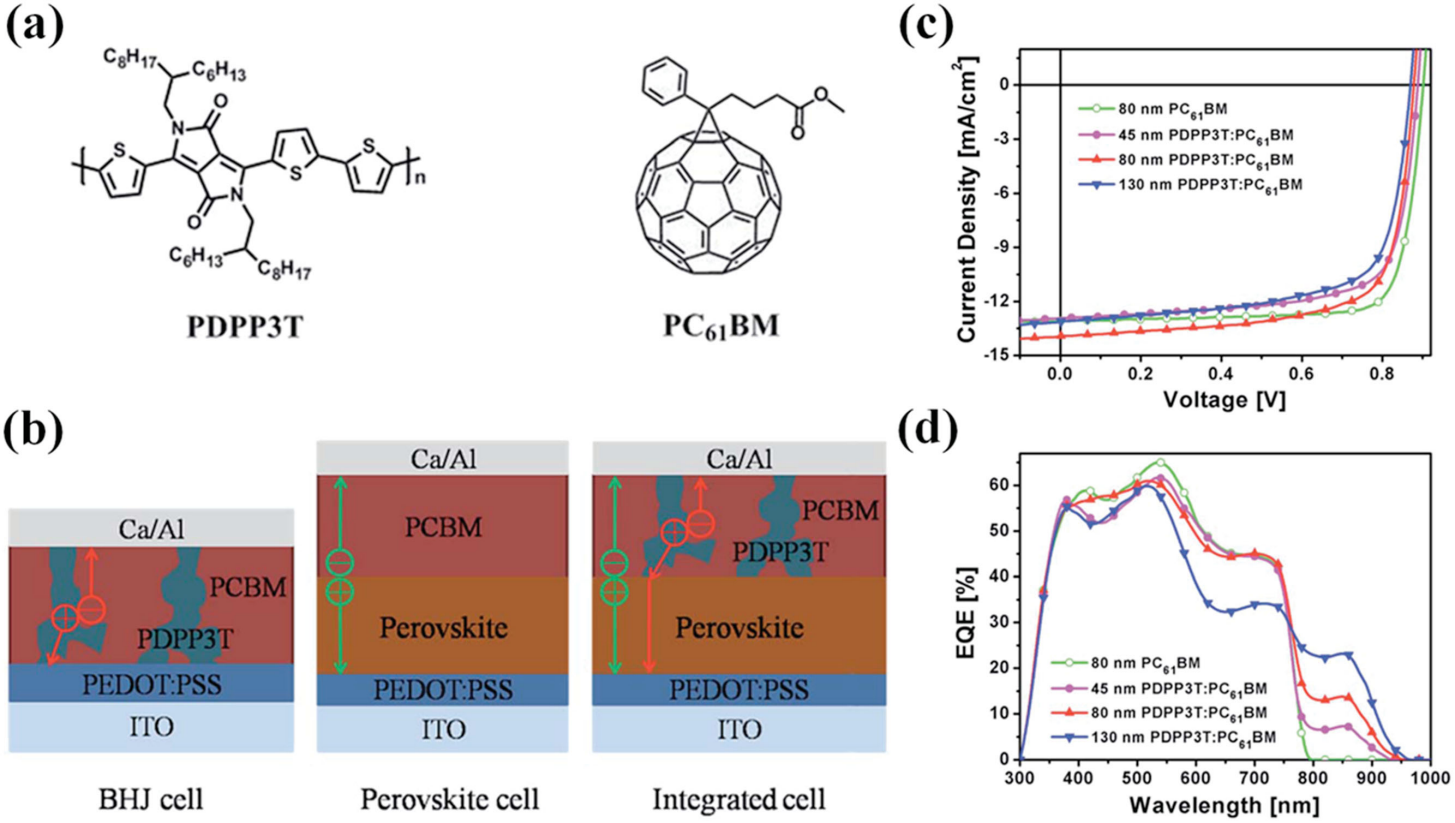
a) Structures of PDPP3T and PC_61_BM; b) structures for BHJ cell, perovskite cell and integrated cell; c) *J–V* curves; d) external quantum efficiency (EQE) spectra. Reproduced with permission.^85^ Copyright 2015, Royal Society of Chemistry.

Zuo et al. expanded the photoresponse of perovskite solar cells by integrating perovskite solar cells with a bulk heterojunction (BHJ) (**Figure** **8**b).^85^ Integrated cells were made by coating a BHJ layer onto CH_3_NH_3_PbI_3_ layer. PCBM is usually used as the acceptor in BHJ solar cells, and also as the ETL in perovskite solar cells. In these device structures, the perovskite layer acts as light absorber and hole transporter. PCBM transports electrons from perovskite and poly(diketopyrrolopyrrole‐terthiophene) (PDPP3T), which is a typical low‐bandgap polymer with an optical bandgap of 1.33 eV. Holes move from PDPP3T to perovskite and are collected by ITO electrode. Both perovskite and PDPP3T contribute to the photocurrent of the integrated cell. The photoresponse was pushed to 970 nm due to the contribution of PDPP3T. *J*_sc_ of the integrated cell exceeded *J*_sc_ of the perovskite cell after optimizing the thickness of BHJ layer (Figure 8c,d). Yang et al. made integrated cells by using DOR3T‐TBDT or PBDTT‐SeDPP as donors in BHJ layer and achieved PCEs of 14.3% and 12.0%, respectively.^86^ The contribution of BHJ to the photocurrent of the integrated cell is the limiting factor. Developing suitable high‐performance BHJ can further enhance the photocurrent and PCE of the integrated cells.

#### 3.5.2 Perovskite/Quantum Dots Integrated Cells

Instead of BHJ cells, QD PV cells have also been integrated with perovskite cells. Seo et al. found that coating a thin layer of CH_3_NH_3_PbI_3_ on PbS QDs to form PbS/CH_3_NH_3_PbI_3_ core/shell structure significantly enhanced *J*_sc_ and PCE of PbS QDs cells.^87^ Etgar et al. made a CH_3_NH_3_PbI_3_ and PbS QDs co‐sensitized cell and got much higher *J*_sc_ than that from single CH_3_NH_3_PbI_3_ and PbS cells.^88^ Hu et al. used PbS QDs as HTM to make perovskite solar cells with a structure of ITO/PbS QDs/CH_3_NH_3_PbI_3_/PC_61_BM/Al.^89^ PbS QDs have a tunable bandgap (≈0.7–2.1 eV) depending on the dot size. PbS QDs with different bandgaps were tried to match the energy level of CH_3_NH_3_PbI_3_. Compared with the cells without PbS QDs, the photoresponse for cells with PbS QDs was expanded due to photocurrent contribution of PbS QDs. The cell with 1.4 eV PbS QDs gave the best PCE of 7.5%.^89^ It is possible to get higher PCE by optimizing device structure or by integrating perovskite cells with other narrow‐bandgap semiconductors like PbSe or SnS etc.

### 3.6 “Switchable” Cells

**Figure 9:**
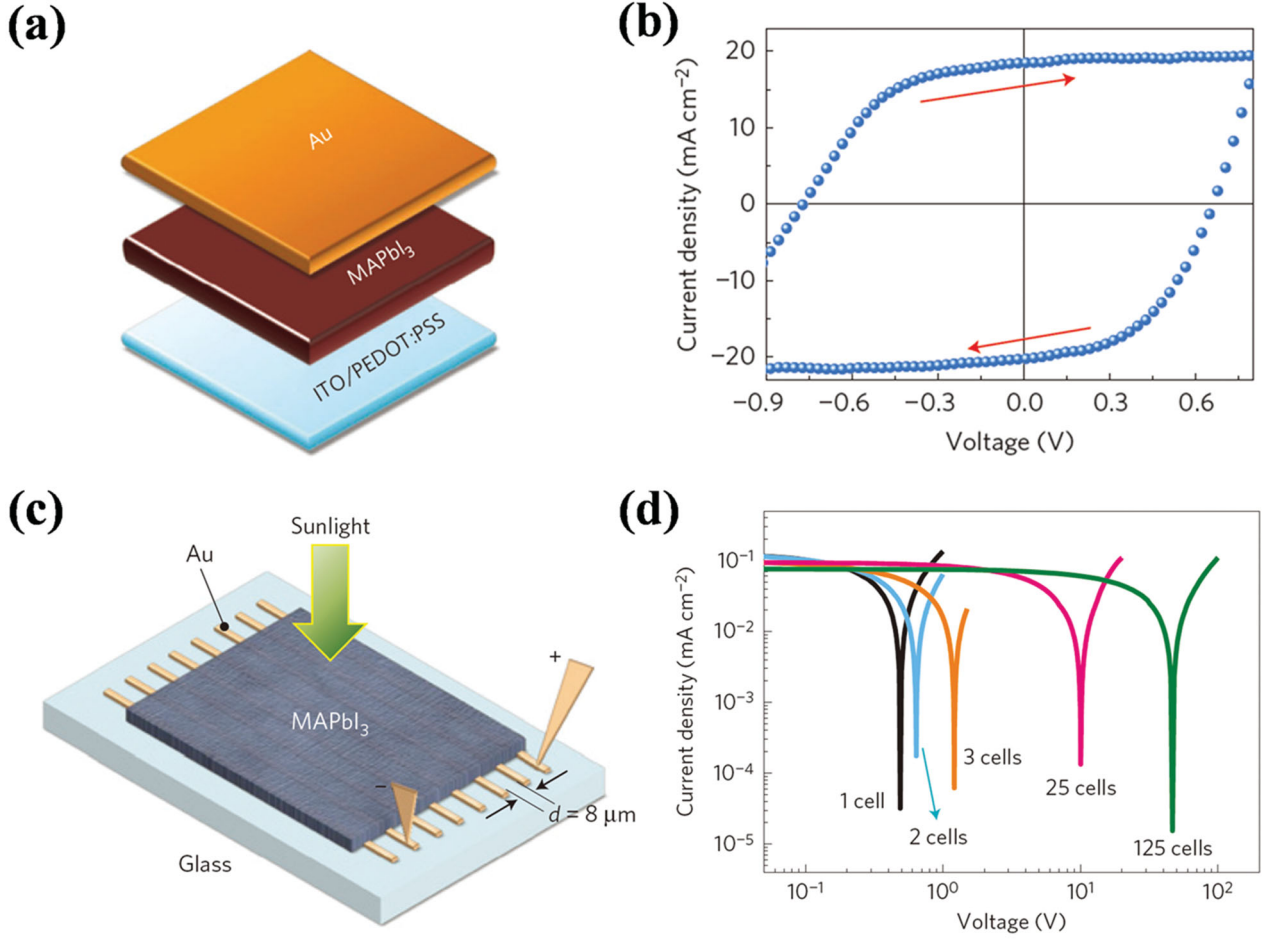
a) Structure for a “Switchable” cell; b) *J–V* curves for a poled cell; c) scheme of the cells on glass with patterned Au electrodes; d) *J–V* curves for cells connected in series. Reproduced with permission.^90^ Copyright 2015, Macmillan Publishers Ltd.

The photocurrent direction for “switchable” cells can be switched by changing the direction of the electric field. Xiao et al. first reported this phenomenon in perovskite solar cells based on CH_3_NH_3_PbI_3_, CH_3_NH_3_PbI_3–*x*_Cl*_x_*, HC(NH_2_)_2_PbI_3_ and CH_3_NH_3_PbBr_3_.^90^ The device structure is shown in **Figure** **9**a. For CH_3_NH_3_PbI_3_ cells, the as‐prepared devices showed low *J*_sc_ (8.5 mA cm^−2^) and *V*_oc_ (0.18 V). The cell gave a *J*_sc_ of 18.6 or –20.1 mA cm^−2^ by applying the electric field from PEDOT to Au (positive poling) or from Au to PEDOT (negative poling) (Figure 9b). After storing the cell for two months, the photocurrent direction kept, suggesting that the poled cell can work as well as those with HTL and ETL. To exploit this feature, they made cells on glass substrate with patterned Au electrodes (Figure 9c). The sub‐cells with a structure of Au/CH_3_NH_3_PbI_3_/Au were connected in series. The non‐poled cell gave no photocurrent. After poling, the cell worked, and a sub‐cell gave a highest *V*_oc_ of 0.88 V. The more the sub‐cells, the higher the *V*_oc_ for the module (Figure 9d). The switchable feature was also observed in cells with a structure of ITO/PEDOT:PSS/CH_3_NH_3_PbI_3_/MoO_3_/Al.^91^

Ion migration was suggested as a possible mechanism behind the switchable feature,[[qv: 3a]],^90^, ^91^, ^92^ although the jury is still out because of the difficulty to perform the classical experiments to prove ion migration (e.g., radioactive iodine tracing, which is problematic because of the gamma emission from the relevant isotope, I^125^).[[qv: 3a]],^92^ Under an applied electric field, the motion of ions in perovskite films would induce a p‐region near one electrode and an n‐region near the other electrode, thus forming a self‐doped p‐i‐n structure,^90^ and similar phenomena have been observed in CuInSe_2_, in light‐emitting electrochemical cells (LECs) and in general, in mixed electronic/ionic conductors.^92^ An opposite electric field can change the p‐i‐n structure into n‐i‐p structure due to the opposite migration of ions. The p‐ and n‐doped regions can act as HTL and ETL, respectively, making the device perform as a common perovskite solar cell.^39^ Thus, changing electric field direction can change photocurrent direction. The observation on changes in work function, composition and morphology during poling supported the ion migration speculation.^90^ The study on transition time for a hole‐only device changing into a diode and the study on interface charge relaxation also supported the viewpoint of ion migration.^91^ Recently, the migration and redistribution of CH_3_NH_3_^+^ ions at room temperature were deduced from photothermal induced resonance (PTIR) measurements.^93^

### 3.7 Single‐Crystal Cells

Single crystal reflects the intrinsic properties of a material. Stoumpos et al. grew single crystals based on tin and lead iodide perovskites and observed reasonable to very high mobilities (>300 up to a few 1000 cm^2^ V^−1^ s^−1^) in CH_3_NH_3_SnI_3_.^94^ CH_3_NH_3_PbI_3_ single crystals show absorption edge at 850 nm, while CH_3_NH_3_PbI_3_ films at 800 nm.^58^, ^94^ The electron/hole diffusion lengths in CH_3_NH_3_PbI_3_ single crystals (>175 μm) are much longer than those in CH_3_NH_3_PbI_3_ films (≈100 nm).^6^, ^58^ CH_3_NH_3_PbI_3_ and CH_3_NH_3_PbBr_3_ single crystals were reported to have very low trap‐state densities comparable to that of best silicon single crystals.^95^

**Figure 10:**
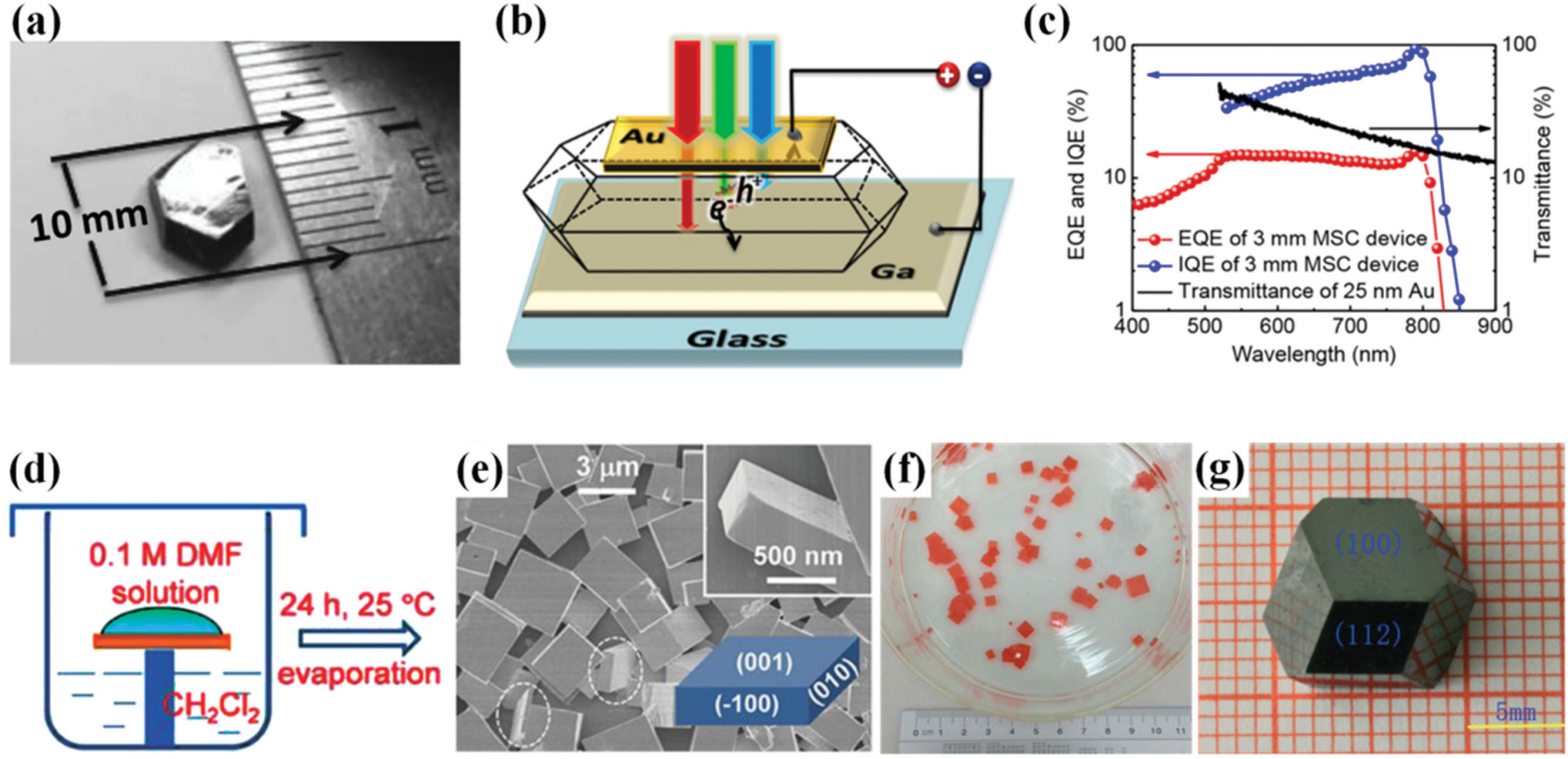
a) A CH_3_NH_3_PbI_3_ single crystal; b) structure of CH_3_NH_3_PbI_3_ single‐crystal solar cell; c) EQE and IQE spectra of such cell; d) scheme of method to prepare CH_3_NH_3_PbBr_3_ single crystals; e) SEM images of CH_3_NH_3_PbBr_3_ single crystals; f) as‐grown CH_3_NH_3_PbBr_3_ single crystals; g) a CH_3_NH_3_PbI_3_ single crystal obtained via a cooling process. a–c) Reproduced with permission.^58^ Copyright 2015, American Association for the Advancement of Science. d,e) Reproduced with permission.^96^ f) Reproduced with permission.^95^ Copyright 2015, American Association for the Advancement of Science. g) Reproduced with permission.^97^ Copyright 2015, Royal Society of Chemistry.

The excellent light absorption and charge transport properties of CH_3_NH_3_PbI_3_ single crystals benefit photovoltaic performance. Dong et al. grew CH_3_NH_3_PbI_3_ single crystals (**Figure** **10**a) using a top‐seeded solution growth method and made solar cells using a 3 mm thick single crystal (Figure 10b).^58^ The external quantum efficiency (EQE) of the solar cell ranged from 12.6% to 15.8% at ≈520–810 nm (Figure 10c). The IQE was nearly 100% at 800 nm. High IQE indicates that electrons generated near Au electrode can move through the whole crystal and be collected by Ga electrode. Long charge carrier diffusion length for CH_3_NH_3_PbI_3_ makes it suitable for X‐ray and γ‐ray sensing. The single crystal device presented an efficiency of 3.9% in radiation sensing.^58^ A solution self‐assembly method (Figure 10d) was used to make CH_3_NH_3_PbBr_3_ single crystals (Figure 10e) for microlasers.^96^ CH_3_NH_3_PbBr_3_ single crystals (Figure 10f) and CH_3_NH_3_PbI_3_ single crystals were prepared via an anti‐solvent vapor‐assisted crystallization.^95^ A 10 mm × 10 mm × 8 mm CH_3_NH_3_PbI_3_ single crystal (Figure 10g) was obtained by using a cooling method.^97^ High‐quality bulk hybrid perovskite single crystals were obtained within minutes by using an inverse temperature crystallization method.^98^

Currently the PCE for single crystal solar cells is still low. The good contact between single crystal and the charge transport layers needs to be realized. Single crystal cells have not reached the performance of cells with polycrystalline films, a feature similar to CdTe and CIGS cells, which distinguishes them from III–V ones (such as GaAs cells), and various reasons have been forwarded for this. The very low barriers between grains may hint that such a difference will remain, but if epitaxial films can be grown, they may yield single crystal cells that can equal thin film ones.

## 4 Applications of Perovskite Solar Cells

### 4.1 Water Photolysis

**Figure 11:**
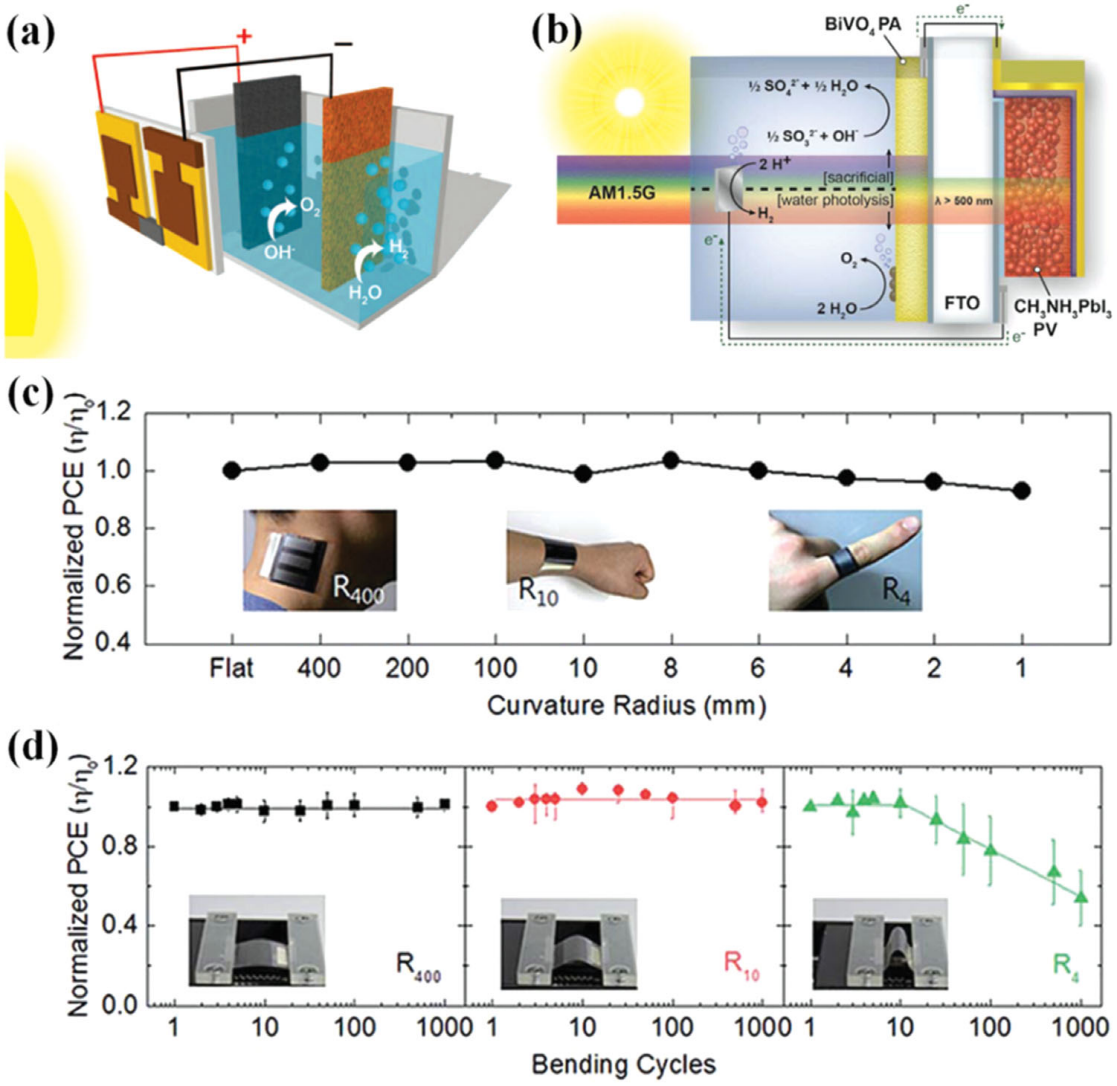
a) Scheme for a water‐splitting device with external power supply; b) scheme for BiVO_4_/CH_3_NH_3_PbI_3_ tandem device; c) normalized PCEs for flexible perovskite solar cells after being bent; d) normalized PCEs for flexible perovskite solar cells as a function of bending cycles with bending radius of 400, 10 and 4 mm, respectively. a) Reproduced with permission.^100^ Copyright 2014, American Association for the Advancement of Science. b) Reproduced with permission.^99^ Copyright 2015, American Chemical Society. c,d) Reproduced with permission.^102^ Copyright 2015, Royal Society of Chemistry.

Water splitting needs a voltage of at least 1.23 V to meet the thermodynamic requirements.^99^ Voltages of 1.8–2.0 V are needed to get acceptable reaction rates for practical applications.^100^ Generally the output voltage for CH_3_NH_3_PbI_3_ solar cells at maximum power point is around 0.9 V, so tandem cells are needed in water photolysis. Luo et al. connected two CH_3_NH_3_PbI_3_ solar cells (outside the electrolyzer vessel) in series to split water (**Figure** **11**a) and achieved a solar‐to‐hydrogen conversion efficiency of 12.3%.^100^ A solar‐to‐hydrogen conversion efficiency of 2.5% was achieved in a perovskite/BiVO_4_ water‐splitting cell, which was made by integrating a BiVO_4_ photoanode with a single junction CH_3_NH_3_PbI_3_ solar cell (Figure 11b).^99^ A solar‐to‐hydrogen conversion efficiency of 2.4% was obtained by integrating a CH_3_NH_3_PbI_3_ solar cell with a Fe_2_O_3_ photoanode.^101^

### 4.2 Wearable Power Source

The popularization of portable electronics drives the development of portable and wearable power supply. The high efficiency and demonstrated flexibility of perovskite solar cells make them suitable for wearable power sources, although safety issue because of Pb toxicity will need to be addressed. Perovskite solar cells can be bent with a bending radius of 1 mm, suggesting that they can be worn on human wrist and even finger (Figure 11c).^102^ The device showed no significant decrease in PCE during 1000 bending cycles with a bending radius around 10 mm (Figure 11d), suggesting that they have potential in practical application. The fiber‐like perovskite solar cells were woven into textiles.^63^, ^64^

### 4.3 Photodetector

As much work has been done on PbX_2_‐based photodetectors, trying Pb halide perovskites is a logical step. Solution‐processed photodetectors based on CH_3_NH_3_PbI_3_ exhibited a large detectivity of 10^14^ Jones and a fast photoresponse, which is better than most of the organic, quantum dot and hybrid photodetectors.^8^ Visible light intensity of 1 pW cm^−2^ can be detected after device noise being reduced via interface engineering and morphology improvement.^103^ Recently, perovskite solar cells were used to detect X‐ray, showing high sensitivity and responsivity.^104^ These results indicate that, if stability issues can be overcome or circumvented, then perovskite solar cells have great potential in photodetector application.

## 5 Conclusions and Perspectives

The ultra‐long electron/hole diffusion lengths, high mobilities, high absorption coefficients and the tunable bandgap of organolead halide perovskite materials drive the research on perovskite solar cells to advance quickly. PCEs over 18% were obtained in different device structures. Flexible and semitransparent devices were fabricated. Perovskite solar cells also find interesting applications in water photolysis, photodetectors and radiation sensing, etc. They not only offer a complementary photovoltaic technology but also provide a platform for developing novel photovoltaic materials and devices.

Nowadays commercial solar cells work under encapsulation, so intrinsic instability (i.e., not due to the atmosphere surrounding the cells) is one of the major concerns, and the other one is the toxicity of Pb, which exists in the cells in a water‐soluble form. Some efforts have been paid to study stability.^105^ Good stability under full sunlight and high temperature was obtained for fully printable mesoscopic perovskite solar cells containing triple mesoporous layers.^65^, ^106^ Replacing part of I^−^ in CH_3_NH_3_PbI_3_ with SCN^−^ can drastically improve the moisture tolerance of the resulting perovskite material.^107^ A crystal‐crosslinking strategy was applied to improve device stability.^108^ Device stability can be further improved by developing novel perovskite materials, device structures, HTL, ETL and electrode materials, and by interface engineering. Highly efficient, stable and environmental‐friendly materials to replace current light absorbers could be found in the near future. Though with drawbacks, perovskite solar cells still show great potential for commercialization because of solution processing, low cost, very high efficiency and various applications. Low‐cost, large‐area fabrication techniques such as printing,^65^ blade coating^109^ and spray coating^110^ are paving the way for commercialization. Hopefully, someday people will find perovskite‐based products in the supermarket.
